# Micro-motors: A motile bacteria based system for liposome cargo transport

**DOI:** 10.1038/srep29369

**Published:** 2016-07-05

**Authors:** Navneet Dogra, Hadi Izadi, T. Kyle Vanderlick

**Affiliations:** 1 Department of Chemical & Environmental Engineering, Yale University, 10 Hillhouse Avenue, New Haven, CT, USA

## Abstract

Biological micro-motors (microorganisms) have potential applications in energy utilization and nanotechnology. However, harnessing the power generated by such motors to execute desired work is extremely difficult. Here, we employ the power of motile bacteria to transport small, large, and giant unilamellar vesicles (SUVs, LUVs, and GUVs). Furthermore, we demonstrate bacteria–bilayer interactions by probing glycolipids inside the model membrane scaffold. Fluorescence Resonance Energy Transfer (FRET) spectroscopic and microscopic methods were utilized for understanding these interactions. We found that motile bacteria could successfully propel SUVs and LUVs with a velocity of 28 μm s^−1^ and 13 μm s^−1^, respectively. GUVs, however, displayed Brownian motion and could not be propelled by attached bacteria. Bacterial velocity decreased with the larger loaded cargo, which agrees with our calculations of loaded bacteria swimming at low Reynolds number.

Mother Nature exhibits numerous forms of micro to nanometer sized motorized machines^1^,^2^. Some of the most intriguing examples are myosin induced muscle contraction^1^,^3^, flagellar motor assisted bacterial propulsion^1^,^4^ and neurotransmitter delivery through synaptic cargo vesicles^5^,^6^. Then at cellular level, single cell organisms such as spermatozoon^7^, bacterium^4^,^8^,^9^ and photosynthetic algae^10^ exhibit motility by responding to the external stimuli. Such machines are very attractive corresponding to their applications in (*i*) micro/nanotechnology, (*ii*) efficient chemical to mechanical energy conversion, (*iii*) engineering better synthetic motors, (*iv*) energy utilization, and (v) lab-on-a-chip devices.

Despite all above seemingly simple examples, it is extremely difficult to harness the power of such natural motors^8^,^10^. Recent attempts have suggested that biocompatibility and use of harsh chemicals are the major reasons of failure^8^,^10^. Nevertheless, remarkable efforts have been made to transport loaded cargo through natural motors by Whitesides^10^, Berg^11^, Gracias^8^, and others^12^,^13^. Several investigators have successfully demonstrated micro-bead (as cargo) transport using motile cells^8^,^10^,^13^. Moreover, cellular motility has been induced through external stimuli such as photo-10, chemo-8, and magneto-taxis^12^. Thanks to all the above efforts, today we have an improved understanding of such biological motors.

Gracias and colleagues have successfully attached 400–600 nm polystyrene beads on motile bacterial surface^8^. Furthermore, tethering soft particles (such as liposomes) with motile bacteria for cargo transport has been recently explored, where bacterial assisted liposomes motility was studied^14^,^15^. Liposomes offer many advantages over micro-beads as a cargo system. They provide (*i*) biocompatibility^16^, (*ii*) biodegradability^16^, (*iii*) and ability to encapsulate drugs^16^,^17^,^18^, genes^19^, enzymes/proteins^17^, and many other molecules^16^,^18^. Since liposomes exhibit both hydrophilic and hydrophobic nature, either kind of molecules can be encapsulated. Another striking advantage of liposomes is that their surface and size (~100 nm to ~20 μm) can be easily tuned^17^. Synaptic vesicle occur inside neurons and are responsible for signal transport, secretion, exocytosis etc^6^,^16^. Recently, synthetic small unilamellar vesicles (SUVs) were put to a great use for mimicking synaptic vesicles^5^. Furthermore, large and giant unilamellar vesicles (LUVs/GUVs) have been explored to mimic the cell membrane surface^20^,^21^,^22^. Combining liposome as a cargo system and natural machines as a carrier is an attractive option for building a better, biocompatible cargo system that could encapsulate drugs, genes or other desired molecules.

We selected motile *Escherichia coli (E. coli*) bacteria as a transporter for liposomes. Bacterial motility is visually appealing and has fascinated scientists for more than a century^4^,^23^. They exhibit unique motility patterns, such as run and tumble, swarm and glide, 180° reverse motion, and frequent decrease in velocity in a chemotaxis buffer^4^,^24^. *E. Coli’s* response to L-aspartate is very interesting. In a concentration gradient of L-aspartate it moves from lower to higher gradient^4^. This motion is usually bidirectional rotation in clockwise (CW) and counterclockwise (CCW) directions^4^. Berg has studied bacterial motility in three dimensional space and suggested the importance of distinguishing random diffusion from chemotactic motility^4^,^24^,^25^. The underlying principle of most motile cells is the conversion of chemical reactions to mechanical energy^1^. In bacteria, an ion-current (H^+^ and Na^+^) driven rotary motor helps bacterial flagella to propel its body^1^,^26^. Certain unique features of motile bacteria such as size at sub-micrometer-scale, precise motility, chemo-attractant/repellent behavior, exhibit response to multiple external stimulus (chemo-, aero-, magneto-, and photo-taxis), are desirable traits from a natural “micro motor”^4^,^9^. From antibody coating^8^,^27^ to chemical probing^10^, there are numerous examples of cargo surface modification. We intend to use a natural procedure that does not put extra strain on bacterial health and motility. Hence, we utilized the inherent property of bacteria by which it binds with gangliosides (glycolipids)^22^,^28^. Gangliosides naturally occur on the eukaryotic cells and have been strongly suggested to act as receptors for bacterial toxins (anchor for pathogen invasion)^28^. Recently, It was found that biomolecular interactions at the membrane surface are region specific and tend to form phase separated domains^21^,^22^. In our case, it is interesting to know if the liposomes prefer specific regions on the bacterial surface.

In our recent work, we investigated motile spermatozoa assisted vesicle transport^7^. We demonstrated that liposome (synthetic vesicles) attached spermatozoa can successfully penetrate through mouse oocytes and deliver cargo^7^. In another study, bacterial interactions were investigated at the liposome bilayer surface^22^. Glucose receptors were covalently attached on the liposomes surface to provide selectivity to protein ligands on bacteria^22^. Moreover, FRET and fluorescence microscopy were utilized for these studies^22^. Herein, we attempt to harness the power of motile bacteria to propel liposomes of multiple sizes (~100 nm to ~20 μm), where liposomes are the cargo system and bacteria performs as a micro motor (Fig. 1). Furthermore, we investigate bacteria-bilayer interactions using FRET spectroscopy and microscopy. To accomplish this, we prepared glycolipids containing liposomes of three sizes (Methods), which provided specificity to the bacterium (Fig. 1A). No further surface functionalization on liposomes or bacteria is required. We have employed optical spectroscopic (UV-Vis, fluorescence, and FRET) and microscopic techniques for investigating these studies.

## Results

### Investigation of bacterial-bilayer interactions using FRET

To investigate the ligand-receptor attachment, we utilized recently reported FRET-based methods, where bacterial interaction with glycolipids probed on the cell membrane surface were analyzed^22^,^29^. FRET only occurs when a donor molecule is in close proximity (typically less than 10 nm) of an acceptor molecule^30^,^31^. Here, we explored FRET for bacterial interactions with glycolipids at the membrane surface. We employed optical spectroscopy and fluorescence microscopy to investigate these interactions. Additionally, we developed a FRET-based microscopy system to identify SUVs interaction at bacterial surface. This assay helped us understand the interactions between oligosaccharide (anchored on the cell membrane surface) and bacteria. Finally we explored SUVs, LUVs and GUVs tethered bacterial motility for transport at micrometer distances.

**Figure 2:**
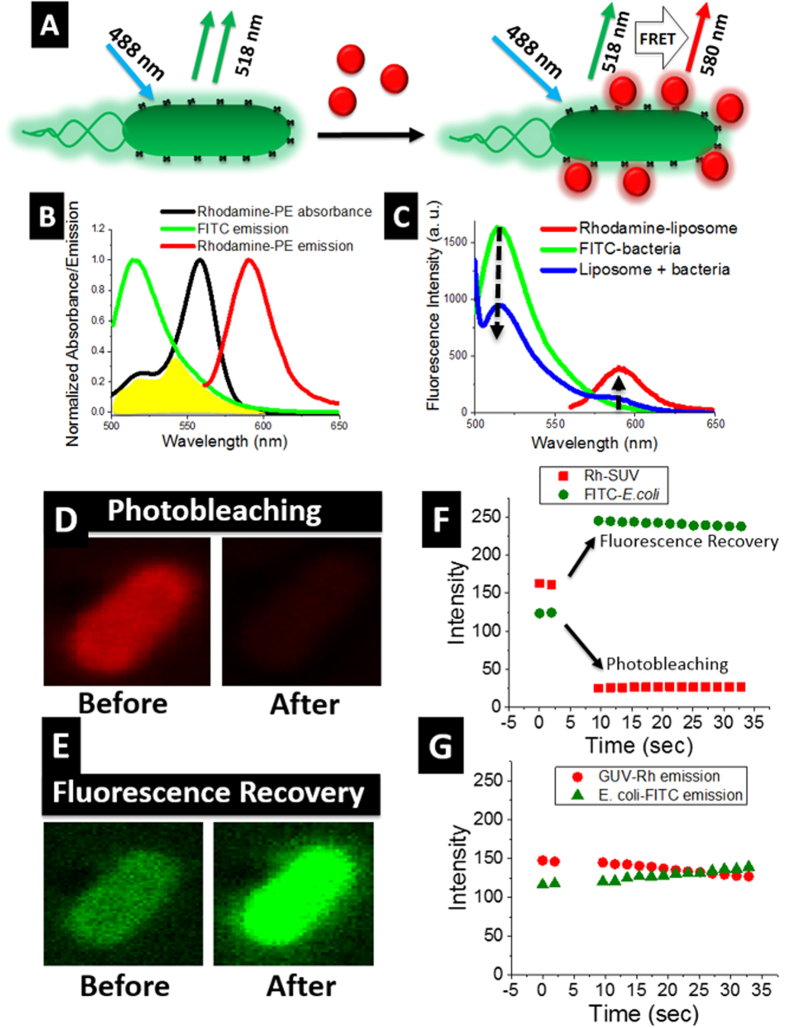
Investigation of bacterial-bilayer interactions using FRET. (**A**) A schematic representation of FITC labeled bacteria (green) and rhodamine labeled SUVs (red). No FRET is observed when nano-liposomes are not in close proximity to bacteria (top left). FRET signal is observed when liposomes attach with bacteria (top right). (**B**) Donor – acceptor spectra for FRET. Spectral overlap between FITC emission (green) and rhodamine absorbance (black) is shaded in yellow. (**C**) FRET is indicated by decrease in the donor (green) fluorescence and an increase in acceptor emission at ~580 nm. Negligible Rh-SUVs emission is observed at λex ~488 (Supplementary Fig. 4S). (**D**) FRET microscopic study of rhodamine photobleaching and (**E**) simultaneous fluorescence recovery of FITC. (**F**) Pixel intensity of photobleaching (rhodamine) and fluorescence recovery (FITC) observed over time. (**G**) Negligible change in FRET intensity is observed in the absence of photobleaching.

Fluorescein isothiocyanate (FITC) and rhodamine are an excellent example of FRET donor acceptor pair (Fig. 2B) and have been frequently used in literature^30^,^31^. By using FITC-labeled bacteria as a donor and rhodamine-labeled liposome (Rh-liposomes) bilayer as an acceptor of fluorescence, we measured FRET signals. Indeed, FRET was apparent as indicated by an increase in acceptor emission while quenching of the donor fluorescence (Fig. 2A,C). We estimated FRET efficiency (*E*) of ~ 40–50% using equation 1 (Methods). In principle, *E* reveals donor and acceptor interactions and is a measure of the distance between the two^32^. In our case, higher *E* is an indication of proximity between Rh-liposomes and FITC–bacteria. Such FRET signals were absent when no Rh-liposomes were present in the solution. Furthermore, to confirm the specificity of bacteria-saccharides interactions, we prepared another set of liposomes, which do not contain oligosaccharides on their surface. Since glycolipids play an important role in bacteria-bilayer interactions, *E. coli* is less likely to interact in the absence of glycolipids on membrane surface. As predicted, we did not observe major FRET signal in this assay (Supplementary Fig. 1). *E* between donor and acceptor dramatically decreased from ~40% to ~5% indicating that FITC–bacteria and Rh–liposomes are not in close proximity. Additionally, this assay also demonstrated that glycolipids are essential for bacterial interactions on bilayer surface^28^.

To visually locate the preference of SUVs interactions at bacterial surface, we observed bacteria-SUVs complexes using FRET microscopy. We followed “acceptor photobleaching” method for these studies (Methods)^31^,^32^. Laser induced photobleaching of rhodamine (acceptor) tagged on liposome surface resulted in a decrease in the rhodamine emission intensity. While simultaneously, we observed a complementary increase (fluorescence recovery) in the FITC (donor) emission (Fig. 2D–F, and Supplementary Movie 6). We did not observe substantial FRET signals when laser induced photobleaching is not applied to the specimen (Fig. 2G). We estimated *E* of ~40–50% from our microscopy data, which was computed to be similar to our spectroscopic *E* data. Furthermore, when we observed the single optical plane (Z-slice) of bacteria - bilayer intersections, specific region where liposomes preferred to interact could be precisely located (Supplementary Fig. 2). Liposomes were segregated on different regions of bacterial membrane as represented by higher fluorescence intensity (of Rh-SUVs) at the edges of bacteria. Moreover, FITC remained around the membrane and did not diffuse in to the cytoplasm of bacteria. These observations showed that both FITC and Rh–SUVs were localized at the bacterial membrane, which further supports the close proximity of SUVs at bacterial surface.

### Monitoring bacteria-liposomes motility

Before observing the motility of liposome–bacteria complex, we studied the motility of unaccompanied bacteria (i.e., bacteria in the absence of attached liposomes). Our bacterial cells exhibited similar characteristics as explained by Berg and Gracias^4^,^8^. The mean velocity of unbound bacteria in a chemotaxis solution has been reported to be ~20–30 μm s^−1^^4^,^8^. Mean velocity (24 μm s^−1^) of bacteria in the present study was analogous to the velocities reported in literature^4^,^8^,^24^. Similarly, our bacteria also moved in CW, CCW and 180° reverse direction (Supplementary Movie 1)^4^.

**Figure 3:**
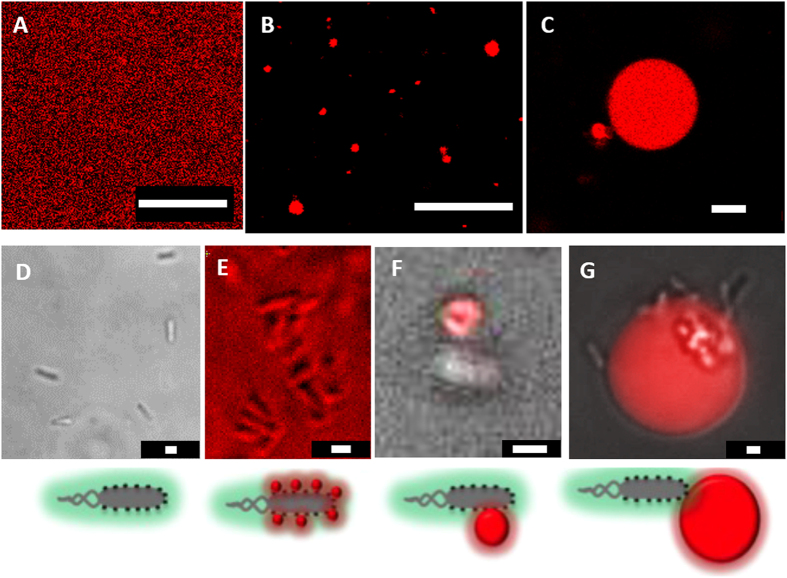
Untethered liposomes and bacteria tethered liposomes microscopic study. Fluorescence micrographs of (**A**) Rh-SUVs, (**B**) Rh-LUVs, and (**C**) Rh-GUVs. (**D**) Optical and fluorescence micrographs of motile bacteria, (**E**) Rh-SUVs attached bacteria (**F**) Rh-LUVs attached bacteria and (**G**) Rh-GUVs attached bacteria. Scale bar for (**A–C)** is 10 μm. Scale bar for (**D–F**) and G is 1 μm.

To test the cargo carrying capability of bacteria, we prepared three sizes (~100–200 nm SUVs, ~1–2 μm LUVs, and ~10–20 μm GUVs) of liposomes (Fig. 3A–C, respectively). Bacteria and liposomes (1 mM) were mixed in a chemotaxis solution at room temperature. The solution was kept stable for at least 4 hours (Methods). This provided enough time and conditions for the glycolipids on liposomes to attach with the ligands on the bacterial surface. This solution was then transferred into a closed chamber and observed under a microscope.

**Figure 4:**
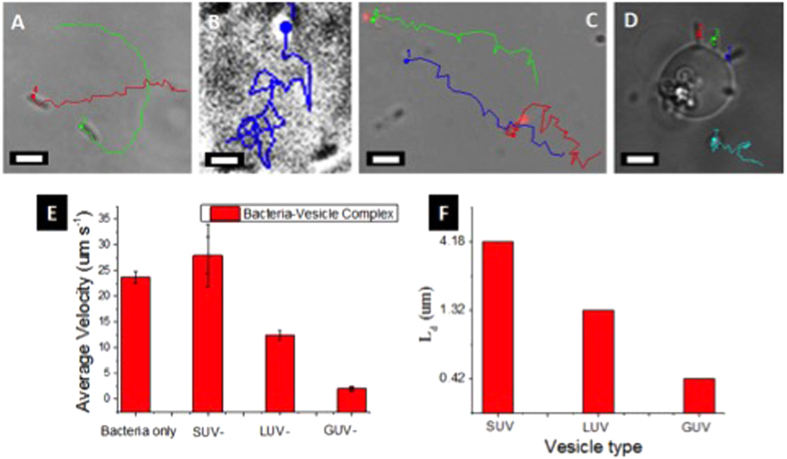
Tracking of liposomes-bacteria motility. (**A**) Tracking of motile bacteria, (**B**) SUVs attached bacteria, (**C**) LUVs attached bacteria, (**D**) GUVs attached bacteria. (**E**) A linear decrease in the velocity of bacterial motility is observed. (**F**) Total calculated diffusion length covered by the vesicles. Scale bar is 2 μm

First, we observed SUVs attached bacterial motility. We prepared Rh-SUVs (~100–200 nm) attached on bacterial surface. SUV–bacteria complexes were studied under a fluorescence microscope. An intense rhodamine emission exhibiting from non-fluorescent bacterial cells indicated that SUVs are attached to the bacterial cells (Fig. 3E). Additionally, our FRET (spectroscopy and microscopy) studies confirmed SUV–bacterial binding (Fig. 2 and Supplementary 2). In our microscopic studies, we did not observe any visible change on the bacterial motility. Similar to the unaccompanied bacteria, SUVs attached bacteria also displayed comparable characteristics (CW, CCW, random tumble and 180° reverse motion) (Supplementary Movie 2 and Fig. 4B). We observed SUV - bacteria for at least 1 hour under a microscope and the cells were motile for the entire duration. Interestingly, SUVs did not significantly affect bacterial motility, as the average velocity of unbound (~24 μm s^−1^) and SUVs bound (~28 μm s^−1^) bacteria were of comparable speeds. Although many bacteria could transport SUVs for upto ~100–200 μm, most bacteria were able to transport SUVs for at least ~50 μm.

Next, we monitored Rh-LUVs (1–2 μm) loaded bacterial motility (Figs 3F, 4C and Supplementary Movie 3). The unique significance of this particular study is that the LUVs and bacteria are of nearly identical sizes. Notably, the average velocity of LUVs loaded bacteria lowered significantly to about half (~13 μm s^−1^) the originally unbound bacteria. This most likely occurred due to larger cargo introducing a higher load on motile bacteria, leading to an increase in the total volume and higher drag on liposome–bacteria complex. We did not observe more than 2 to 3 LUVs attached to single bacteria. The reason behind a selective number of bacteria binding to the liposomes is restricted by the total surface area of bacteria/liposomes and the number of receptors (glycolipids) on liposome surface (~33% glycolipids). After the attachment, bacteria were motile and could transport LUVs for at least ~50 μm.

Finally, we prepared GUVs attached bacteria. Here, the liposome size is ~5–10 times larger than the size of bacteria. To our surprise, the average velocity of GUV bound bacteria was lowered by ~10 folds to ~2 μm s^−1^ (Figs 3G and 4D, and Supplementary Movie 4). We think that this minimal motility is most likely due to the Brownian motion of GUVs and has little contribution from bacterial motility. To understand if there is any liposome deformation or adhesion to the cover-slip, we carefully observed the three dimensional structure of bacteria attached GUV (Supplementary Fig. 3). We found minimal deformation and the liposome was found to be in its original, intact shape (Supplementary Fig. 3). There is a need of higher bacterial cells attached (higher collaborative forces) to the liposome surface for GUVs transport. This is our ongoing endeavor in this direction. We frequently observed a few bacteria rotating in a circular motion (Supplementary Movie 5), which occurred due to bacterial adhesion to the cover slip surface. This phenomenon has been observed and reported previously, where van der Waals forces and flagella related adhesion were considered as the major reason of circular trajectories^8^.

### Motility versus diffusion

It is important to distinguish the distance covered by liposomes through diffusion versus bacterial propulsion. The diffusion lengths travelled by liposomes can be calculated by using Stokes–Einstein–Sutherland equation (*D* = *k*_*B*_*T*/(6πηR) and diffusion length equation (*L*_*d*_ = √(4*Dt*)) (Methods & Supplementary Table 1)^13^. The total diffusion length of the liposomes was calculated to be ~7–8 times smaller (0.42 μm for GUVs, 1.32 μm for LUVs, and 4.18 μm for SUVs) than the observed displacement of bacteria loaded liposomes (Fig. 4E). This confirms that bacteria are responsible for the liposome propulsion and random diffusion does not contribute to the longer distances travelled by liposomes. Purcell’s seminal study about bacterial propulsion at low Reynolds number has been instrumental in understanding the effect of viscous drag on bacterial surface^33^. At low Reynolds number, particle velocity depends linearly on viscous drag^33^, i.e., as the particle becomes larger, the velocity should lower linearly. However, as reported recently, the viscous drag introduced by the attached bead (3 μm) on the cell surface (10 μm) swimming at a velocity of 100–200 μm s^−1^, only contributes to a fraction of total drag force being applied on a cell surface^10^. In our case however, SUVs and LUVs attached on the bacterial surface are similar scaled complexes to above-mentioned studies. Nonetheless, when GUVs (~10 μm) are attached on the bacterial surface (~2 μm), it is the GUV’s drag force that dominates the system and introduces higher overall drag. A theoretical drag force can be computed using *F*_*drag*_ = *6πηRU* (Methods & Supplementary Table 1). At a speed of 25 μm s^−1^, the viscous drag introduced by SUVs and LUVs is 0.024 pN and 0.24 pN respectively, which are much lower than the drag experienced by bacteria (0.47 pN). However, GUVs introduce a drag of about 5 times higher (2.4 pN) than that of bacteria, which is highly likely to contribute to the lower velocity for larger load on bacteria.

## Discussion

We have investigated bacteria-bilayer interactions by using glycolipids on the model membrane scaffold. Furthermore, we demonstrated motile *E. coli* bacteria assisted propulsion of liposomes. Our microscopic observations demonstrate that only 30–40% of liposomes interacted with bacteria. Although, we intended to functionalize all liposome particles with glycolipids (for every liposome preparation we used following lipid ratio: DOPC:cholestrol:ganglioside::1:1:1 + 5ul Rh-PE), we think that disproportionate bacterial binding could be due to the irregular distribution of glycolipids (gangliosides) inside model membrane in different liposomes. In our confocal fluorescence microscopic observation we found that glycolipid distribution inside the membrane is uneven in different liposomes particles (Supplementary Fig. 5). We have confirmed in our FRET studies that liposomes with no glycolipids on its surface do not interact with bacteria. This study further demonstrates that glycolipid distribution is directly related with bacterial attachment on single liposome particle. Another important point to mention here is that bacteria are not motile in the absence of flagella. The flagella of *E. coli* are extremely important tool for its motility. We have mentioned in our methods section that bacteria were cultured at 32 °C. Which is a very important parameter for motile bacteria growth. The bacteria grown in our lab at 37 °C were not motile as higher temperature inhibits the flagellar growth^4^.

Present study has many striking potential applications such as: (*i*) power utilization (single cell provides the power to propel cargo), (*ii*) cargo transport at micro distances, (*iii*) loaded liposomes can be utilized as delivery system, (i*v*) high efficiency of chemical to mechanical energy in a micro-motor, and (v) fundamental understanding of bacterial invasion on cell bilayer. Nevertheless, this approach does have its limitations. (*i*) Bacteria are time sensitive machines, as their motility lowers with duration (motility usually lasts for 1–2 weeks). (*ii*) Bacterial motility is dependent on experimental conditions (solution, temperature, etc.). However, we believe that with newer developments in genetic engineering, most hindrances can be resolved in the near future. On a final note, this is our attempt for interactions of soft particles (vesicles) at bacterial cell membrane surface. Our soft particles induce less strain on the cell membrane (as they conform to the membrane shape), and are biodegradable. Further work requires release of the attached cargo at a designated system, place and distance. The use of collaborative bacterial forces to propel larger cargo and enzymatic action to release the cargo are our ongoing endeavor.

## Methods

Reagents. 1, 2-dioleoyl-sn-glycero-3-phosphocholine (DOPC), cholesterol (chol), total gangliosides (gang) were purchased from Avanti Polar Lipids (Alabaster, AL). 1,2-dihexadecanoyl-sn-glycero-3-phosphoethanolamine Lissamine rhodamine B (Rh-PE) were purchased from Invitrogen Molecular Probes. Fluorescein isothiocyanate (FITC) and α-Methyl-DL-aspartic acid were purchased from Sigma-Aldrich. Chloroform, methanol were purchased from Fisher Scientific. Sodium Hydroxide (NaOH), Dimethyl sulfoxide (DMSO) were purchased from Sigma Aldrich.

### Vesicle Preparation

Small Unilamellar Vesicles (SUVs). (100–200 nm). SUVs were prepared by following our previously published work^22^. A mixture of lipids in a desired ratio (dopc:chol:gang::1:1:1 + 5 μl Rh-PE) was dissolved in chloroform in a round bottom flask. Lipid solution was dried under vacuum for at least 4 hours. The resulting thin film was hydrolyzed by adding nano pure water to bring the final lipid concentration to 1 mM. The resulting suspension was sonicated (Virsonic ultrasound cell distrupter) at 75 °C for 20 minutes. Immediately, the solution was filtered through 0.22 μm syringe filter (purchased from Millipore). The resultant optically clear vesicle solution is cooled to room temperature and kept at 4 °C for later use.

Large Unilamellar Vesicles (LUVs). (1–2 μm diameter). We prepared GUVs by following electroformation method^34^. A mixture of lipids in a desired ratio (dopc:chol:gang::1:1:1 + 5 μl Rh-PE) was dissolved in chloroform in a round bottom flask. Using a syringe, about 50 μl mixture of Lipid stock solution in chloroform were deposited drop wise on platinum wires in a homemade electroformation chamber. The platinum wires were dried under vacuum for at least 4 hours. The chamber was tightly closed by using BSA coated slides. Preheated nano pure water was added using a syringe to the electroformation chamber to make the final concentration to 1 mM. The chamber was transferred to a preheated at 70 °C oven so that all lipids stay in fluid phase. An electric field of 3.0 V a.c. was applied across the platinum wires at 10 Hz for 30 min, 3.0 Hz for 15 min, 1.0 Hz for 7 min and 0.5 Hz for 7 min. Vesicles were then carefully removed from the chamber by using a syringe. The vesicle solution was cooled down to room temperature and kept in the refrigerator at 4 °C.

Giant Unilamellar Vesicles (GUVs). (~10–20 μm diameter)^20^,^21^,^22^. We followed a previously published literature for preparing GUVs^17^,^22^. Briefly, a mixture of lipids (dopc:chol:gang::1:1:1 + 5 μl Rh-PE) was dissolved in 980 μl of chloroform and 200 ul of methanol in a round bottom flask. 7 ml of nano pure water was added through the sides of the flask by minimal disturbance to the solution. The organic solvent was removed at 40 °C by rotary evaporator under reduced pressure. After 2–3 minutes, an opalescent fluid was obtained. This fluid can be easily visualized through naked eyes. The vesicle solution was cooled down to room temperature and kept in the refrigerator at 4 °C.

### *E. coli* growth

*E. coli* RP437 was provided by coli genetic stock center (CGSC) *E. coli* Genetic Resources, at Yale University. Bacteria cultures were grown overnight at 32 °C in a tryptone broth (TB) in a 125 ml shake flask in an incubator shaker. *E.coli* grown at higher temperature were found to be of lower motility (higher temperature inhibit flagella growth). The overnight culture was transferred to an Erlenmeyer flask to an optical density of ~0.05 (at 600 nm). The cells were re-suspended in chemotaxis buffer (CB).

### Fluorescence labeling

Bacteria were washed with PBS three times before labeling with FITC. Cells were incubated in phosphate buffer and the pH was maintained to 9.2 by adding NaOH solution drop wise to the buffer. Briefly, FITC in DMSO (1 mg/ml) was added to the *E. coli* solution at 25 °C for 4 hours. The unlabeled FITC was separated by centrifuging at 800 rpm for 60 seconds repetitions until a clear supernatant is achieved. To confirm successful FITC labeling, the bacteria are visualized under a fluorescence microscope. The motility buffer consisted of 10 mM PO4^3^−, 0.1 mM EDTA, (pH 7.3), and 1 mM of α-Methyl-DL-aspartic acid.

### FRET spectroscopy

In spectroscopy assay, we utilized FITC and rhodamine as donor acceptor pair respectively. The excitation wavelength for FITC (donor) was at 488 nm. We observe a major emission spectra associated with FITC emission at λmax ~518 nm. As we add small aliquots of rhodamine tagged liposomes a decrease in FITC emission and a slight increase in rhodamine emission is observed. This is an indication of nano-liposomes interacting on the bacterial surface. As bacteria interacts with glycolipids attached on the liposome surface, FITC molecules tagged on bacteria approach rhodamine molecules on the liposome surface. All fluorescence spectra were taken using Hitachi fluorescence spectrometer (F-4500) and collected using FL-solutions software. All UV-Vis absorption spectra were taken using Agilent cary-100 UV-Vis spectrometer and collected using cary winUV software. The spectra were further normalized using freely available (for schools) software “specwin” (http://www.effemm2.de/spekwin/spekwin_beschr_en.html).

### Microscopy

Nikon Eclipse TE200 inverted fluorescence microscope equipped with 40X DIC objective with attached video camera (Q imaging RETIGA-SRV fast-1394) was used for bacterial tracking and fluorescence microscopy.

### FRET microscopy

We used Zeiss LSM 710 Duo NLO/Multiphoton Microscope, equipped with 488 and 561 nm laser (63x oil immersion objective lens) for analyzing FRET between SUVs and bacteria. In the present method donor emission is measured before and after the photobleaching of acceptor. We measured FITC (donor) emission before and after the photobleaching of rhodamine (acceptor). We used high intensity laser to photobleach (λex at ~561 nm) rhodamine after 10 seconds of sample exposure to normal laser intensity. Simultaneously, FITC emission (λex ~488 nm) at 518 nm is observed. Finally, pixel intensity is obtained from the microscopic image. No further image modification was carried out.

### FRET efficiency (*E*)

*E* is calculated using following formula^31^.


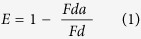


where *Fda* and *Fd* are donor emission intensities in the presence and absence of acceptor respectively.

### Image processing

The bacterial motility movies were processed in Image J, using a particle tracking plugin (free software and plugin available from NIH, (http://rsbweb.nih.gov/ij/). Plugin “manual tracking” was used for tracking bacterial motility. The bacterial motility in all movies were visualized using the following procedure: Manual tracking > Drawing > Overlay Dots & Lines. All movies were processed to “Enhance contrast” for better visibility.

### Estimation of Diffusion coefficient (*D*), diffusion length (*Ld*), and Drag Force (*F_drag_*)

The diffusion lengths travelled by liposomes were calculated by using

Stokes–Einstein–Sutherland equation:^12^,^13^


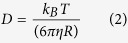


where *D* is the diffusion coefficient, *kB* is Boltzmann’s constant, *T* is the absolute temperature of the solution, *η* is the dynamic viscosity of water, and *R* is the radius of the particle

Diffusion length equation:^12^,^13^


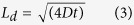


where *D* is the diffusion coefficient and t = 1 s is the time.

Drag force can be computed using the following equation:


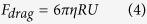


where *F_drag_* is the drag force and *U* is the velocity. *η* is the dynamic viscosity of water, and *R* is the radius of the particle.

## Additional Information

**How to cite this article**: Dogra, N. *et al*. Micro-motors: A motile bacteria based system for liposome cargo transport. *Sci. Rep.* **6**, 29369; doi: 10.1038/srep29369 (2016).

## Supplementary Material

Supplementary Information

Supplementary Information

Supplementary Information

Supplementary Information

Supplementary Information

Supplementary Information

Supplementary Information

## Figures and Tables

**Figure 1:**
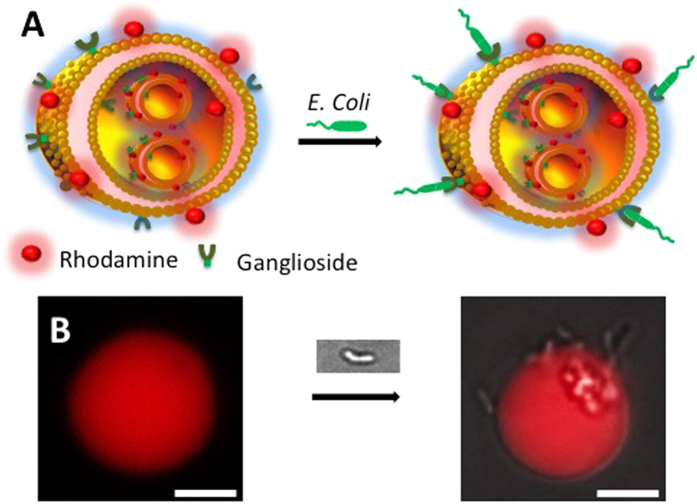
Bacteria-GUV interaction. (**A**) A schematic representation of surface functionalized liposomes (GUVs) before (left) and after (right) bacteria attachment. (**B**) Fluorescence micrographs of GUVs before (left) and after (right) bacterial attachment. Scale bar is 5 μm.
