# Supplementary material for: Micro-motors: A motile bacteria based system for liposome cargo transport
Source: Sci Rep. 2016 Jul 5;6:29369. doi: 10.1038/srep29369 (PMC4932553; doi:10.1038/srep29369)
Supplement: Supplementary Information [file srep29369-s7.pdf]

Supporting Materials

Micro-motors: A motile bacteria based system for liposome cargo transport

Navneet Dogra, Hadi Izadi & T. Kyle Vanderlick*

Department of Chemical & Environmental Engineering, Yale University, 10 Hillhouse Avenue, New Haven, CT, USA

Supplementary Movie 1. Tracking of bacteria cells under an optical microscope.

Supplementary Movie 2. Tracking of SUV loaded bacterial cells under a fluorescence microscope. It must be noted that the cells are unlabeled and the fluorescence is coming only from rhodamine labeled SUVs.

Supplementary Movie 3. Tracking of LUV loaded bacterial cells under a fluorescence microscope. LUVs are rhodamine labeled. Bacteria are non-fluorescence.

Supplementary Movie 4. Tracking of GUV loaded bacterial cells under an optical microscope.

Supplementary Movie 5. Surface adhered bacterial cell.

Supplementary Movie 6. FRET measurements of Rh-SUVs attached FITC stained *E. coli*. When Rh-SUVs are photobleached, fluorescence recovery from FITC is observed.

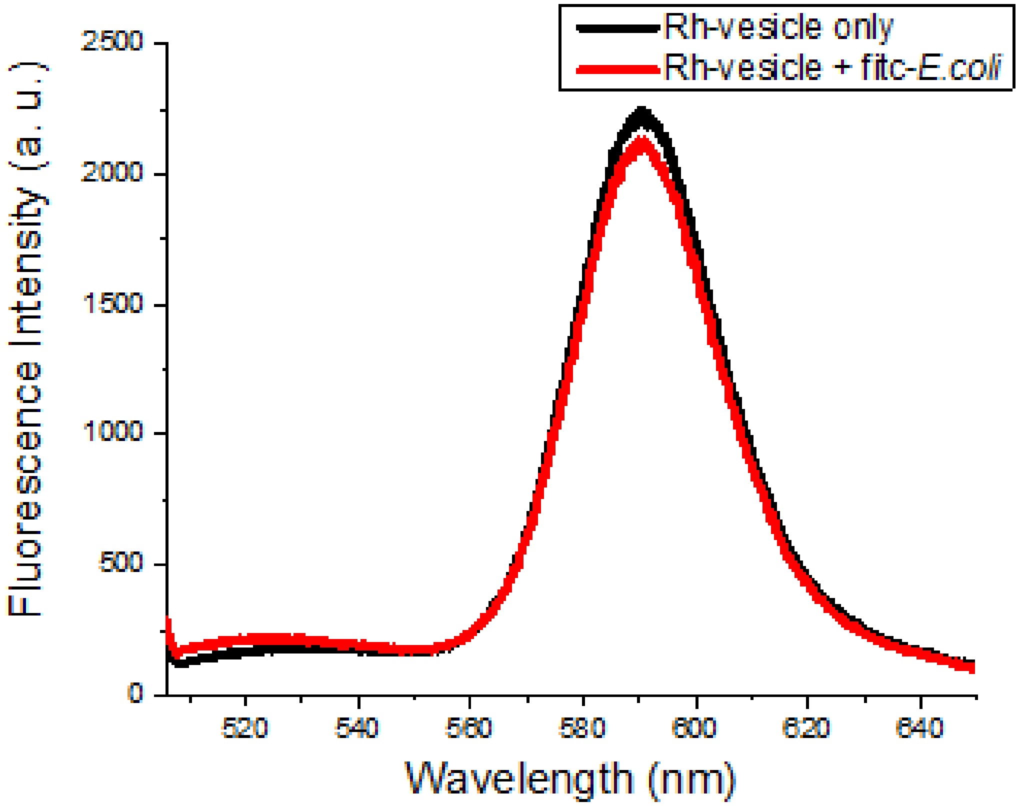

Supplementary figure 1. No Glycolipids is probed on rhodamine-vesicles shows negligible FRET. Above figure shows before (black) and after (red) bacteria addition to the vesicle solution.

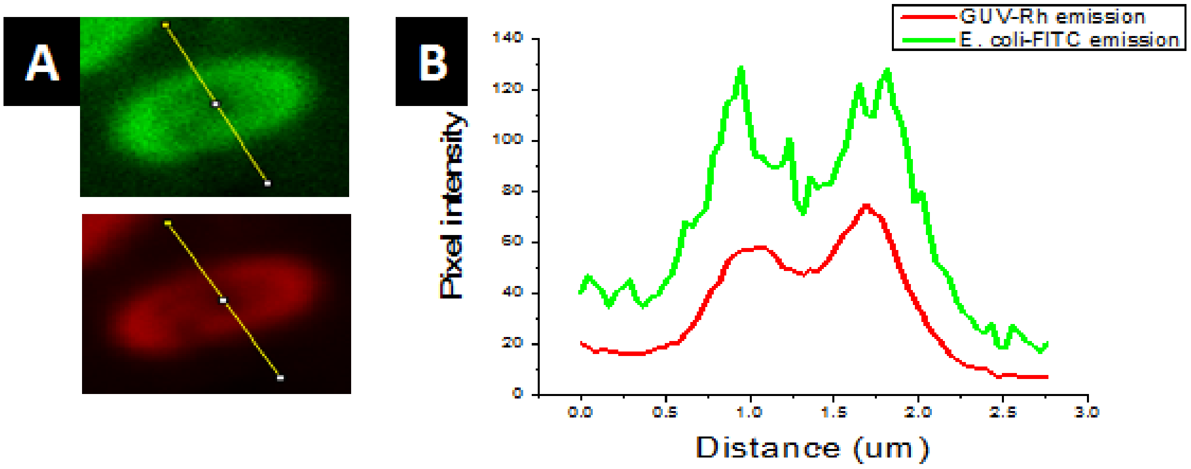

Supplementary figure 2. Single optical plane (Z-slice) image of FITC-bacteria (A green) and Rhodamine-SUVs attached to bacteria (A red). (B) Measured pixel intensity of green and red fluorescence from A.

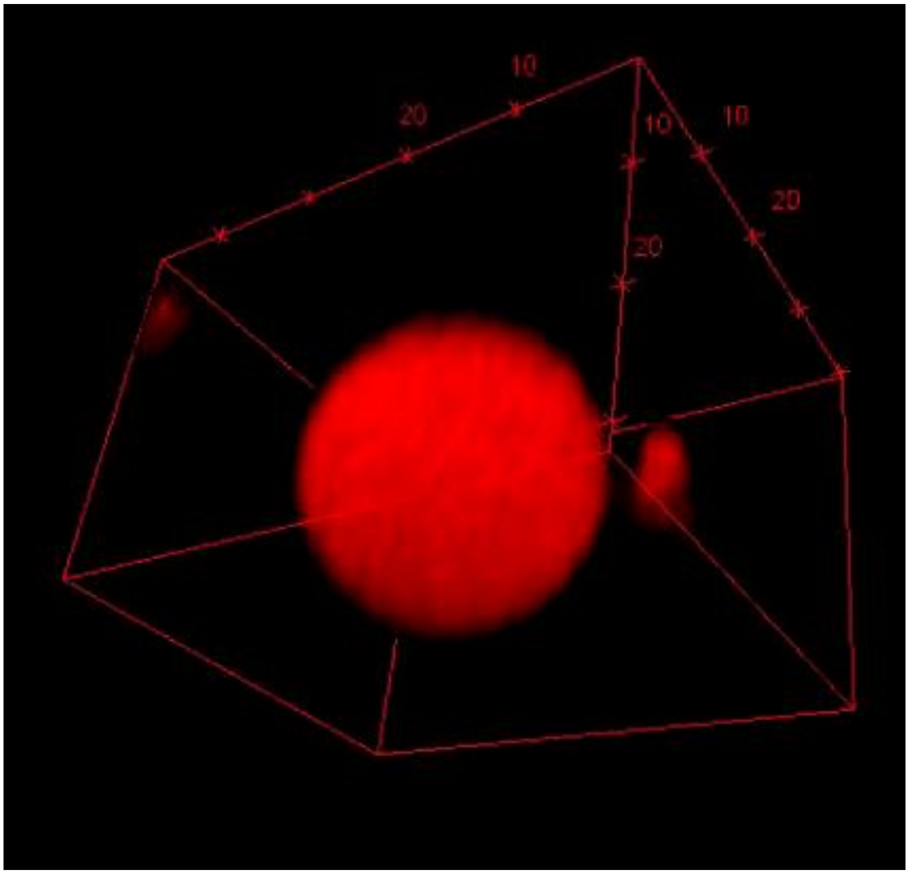

Supplementary Figure 3. A three dimensional recreation of GUV to observe major deformations in the vesicle.

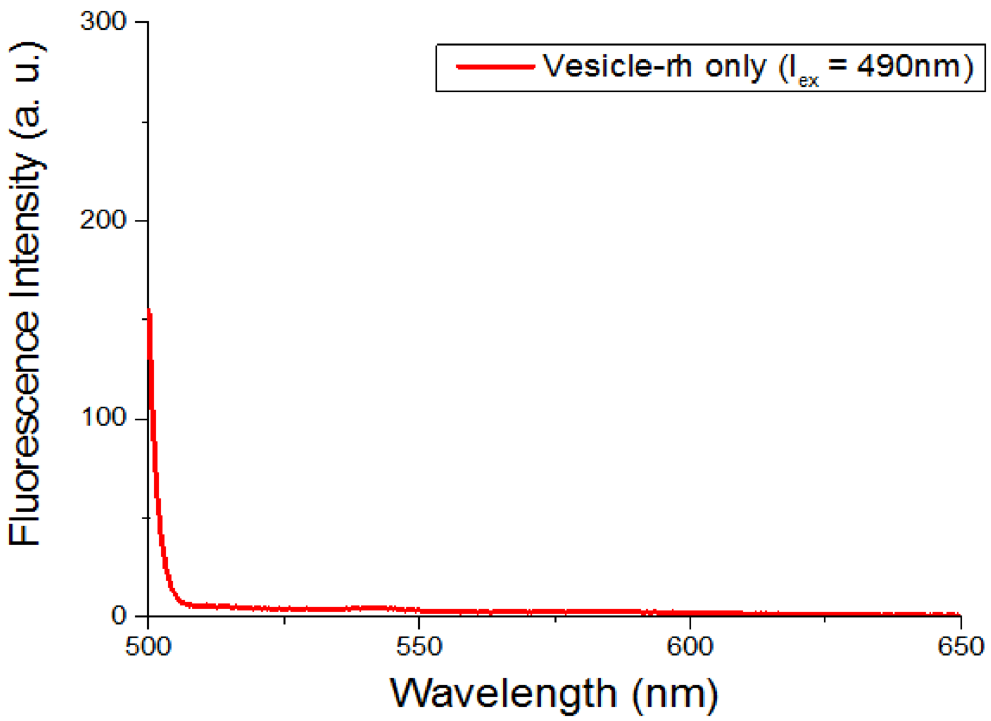

Supplementary Figure 4. Rhodamine vesicles excited at 490 nm exhibit negligible fluorescence.

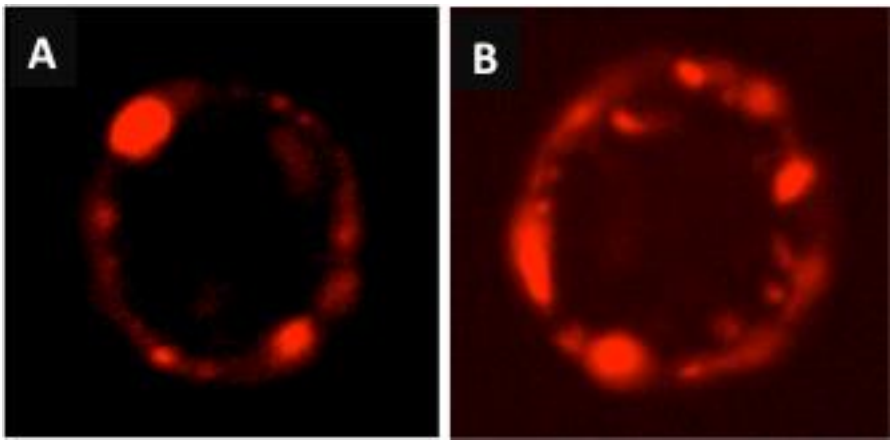

Supplementary Figure 5: Fluorescence microscopic observation of single optical plane (z-slice) taken for two different GUVs. (A), (B) Uneven distribution of glycolipids can be visualized as the non-fluorescent domains in the membrane.

Particle type/size	Diffusion Coeff. (D) m^2 / s	Diffusion Length (L_D) µm	Drag Force (Fdrag) pN
~10 µm GUVs	$4.37x10^{-14}$ m^2 / s	0.42 µm	2.4 pN
~1 µm LUVs	$4.37x10^{-13}$ m^2 / s	1.32 µm	0.24 pN
~100 nm SUVs	$4.37x10^{-12}$ m^2 / s	4.18 µm	0.024 pN
~2 µm Bacteria	$2.18x10^{-13}$ m^2 / s	0.93 µm	0.47 pN

Supplementary Table 1. Theoretical calculations. A comparison of Diffusion coefficient (D), Diffusion length (*Ld*), and Drag Force (F_{drag}) based on the particle size.

Velocity statistics: For every single velocity measurement, we tracked three individual bacteria and an average velocity value was used (Table 1S). For every measurement fresh bacteria were used within first week of culture. The bacteria were motile for at least 2 weeks. Image J particle tracking system was used for velocity calculation and analysis.

sample	Velocity	Average Velocity
Bacteria only	24.87	23.8
	22.76	
	23.74	
Bacteria-SUV	24.48	28.18
	29.03	
	31.05	
Bacteria-LUV	11.52	12.48
	13.41	
	12.5	
Bacteria-GUV	1.56	2.05
	2.23	
	2.35	

Supplementary Table 2.: Average velocity of bacteria is calculated by tracking three individual bacteria.
